# 

**Published:** 1920-09

**Authors:** 


					CONTENTS. Page
^*nnine-Dropsy as a Food-Deficiency Disease.
By J. A. Nixon,
C.M.G., M.D., F.R.C.P.
137
Vesical Calculus in a Urinary Typhoid Carrier.
By Duncan Wood,
F.R.C.S ...
148
Poisoning by "Mustard" Gas.
By E. H. E. Stack, F.R.C.S.,
Capt., R.A.M.C. (T.F.), Carey F. Coombs, M.D., F.R.C.P., Capt.,
R.A.M.C. (T.F.), and R. Rolfe, B.A., M.B., B.Ch. Cantab.
151
Progress of the Medical Sciences :
Medi
G. Parker, M.A., M.D. Cantab. ...
162
Surgery.
J. Lacy Firth, M.S., M.D.
165
Reviews of Books
170
Editorial
Notes
178
Library of the Bristol Medico-Chirurgical Society   182
Obituary
184
David E. Bernard.
Lionel Shingleton Smith.
^?Cal Medical Notes
187

				

